# Non-fatal self-harm in Scottish military veterans: a retrospective cohort study of 57,000 veterans and 173,000 matched non-veterans

**DOI:** 10.1007/s00127-018-1588-9

**Published:** 2018-08-25

**Authors:** Beverly P. Bergman, Daniel F. Mackay, Daniel J. Smith, Jill P. Pell

**Affiliations:** Institute of Health and Wellbeing, Public Health and Health Policy, University of Glasgow, 1 Lilybank Gardens, Glasgow, G12 8RZ UK

**Keywords:** Military veterans, Self-harm, Early service leavers, Retrospective cohort studies, Comorbidity

## Abstract

**Purpose:**

Although suicide risk in veterans has been widely studied, there is little information on the risk of non-fatal self-harm in this population. We used data from the Scottish Veterans Health Study to conduct an epidemiological analysis of self-harm in veterans, in comparison with people who have never served.

**Methods:**

We conducted a retrospective, 30-year cohort study of 56,205 veterans born 1945–1985, and 172,741 people with no record of military service, and used Cox proportional hazard models to examine the association between veteran status and cumulative risk of non-fatal self-harm, overall and stratified by birth cohort, sex and length of service. We also examined mental and physical comorbidities, and association of suicide with prior self-harm.

**Results:**

There were 1620 (2.90%) first episodes of self-harm in veterans, compared with 4212 (2.45%) in non-veterans. The difference was statistically significant overall (unadjusted HR 1.27, 95% CI 1.21–1.35, *p* < 0.001). The risk was highest in the oldest veterans, and in the early service leavers who failed to complete initial training (unadjusted HR 1.69, 95% CI 1.50–1.91, *p* < 0.001). The risk reduced with longer service and in the intermediate birth cohorts but has increased again in the youngest cohort.

**Conclusions:**

The highest risk of non-fatal self-harm was in veterans with the shortest service, especially those who did not complete training or minimum engagement, and in the oldest birth cohorts, whilst those who had served the longest were at reduced risk. The risk has increased again in the youngest veterans, and further study of this subgroup is indicated.

## Introduction

In recent years provision for the health and welfare needs of veterans has assumed increasing importance [1], and it is acknowledged that the planning of effective support has to be underpinned by sound data [2]. The Scottish Veterans Health Study was conceived in order to demonstrate areas where the health of military veterans in Scotland differed fom that of the wider non-veteran population, and thereby act inter alia as a needs assessment to identify where specific support or intervention may be required [3].

A number of studies have examined suicide in UK military veterans [4–7], and other studies have looked at suicidal ideation in recently deployed US veterans [8], but to date there has been little research on the longer term risks of non-fatal self-harm in the overall veteran population [9], particularly in the UK where risks are known to differ from the US military population [10]. This represents an important knowledge gap since self-harm is a recognised risk factor for subsequent suicide [11] and may act as a pointer to unmet welfare needs.

Non-fatal self-harm encompasses both failed suicide and events having no suicidal intent; conversely, suicide may encompass events where a fatal outcome was not intended but the lethality of the method was not appreciated. Intent is rarely documented and, therefore, at a population level, it is necessary to consider all non-fatal episodes together.

We used data from the Scottish Veterans Health Study, a retrospective cohort study of 57,000 veterans and 173,000 matched non-veterans, to examine the epidemiology of non-fatal self-harm over a 30-year period in a broad national cohort of veterans in Scotland, irrespective of combat or deployment, in comparison with people having no record of military service. We investigated the association between suicide and a previous episode of self harm. We also examined a range of comorbidities and explored potential risk factors for self-harm so far as was feasible within the available retrospective dataset.

## Methods

The Scottish Veterans Health Study is a retrospective cohort study of all 56,570 military veterans in Scotland who were born between 1945 and 1985, who were resident in Scotland and were registered with the National Health Service (NHS) Scotland both before and after service, and a comparison group of 172,753 individuals with no record of service (‘non-veterans’), matched 3:1 for age, sex and postcode sector of residence (mean population 5,000). We used Scottish linked health records (which cover all individuals registered with NHS Scotland) and mortality data to compare long-term risk of suicide and major self-harm in veterans and non-veterans, obtaining a single retrospective data download as at 31 December 2012. The study cohort and methods have been described in detail previously [12], and the findings in respect of suicide have recently been published [7]. This paper examines non-fatal self-harm leading to hospitalisation, encompassing both non-suicidal intent and failed suicide. Baseline demographic data, including veteran status and dates of entering and leaving military service for veterans, were provided by NHS electronic registration records. Reservists with no regular military service were excluded as their veteran status cannot be determined from the NHS registration record. The data were linked at an individual level to routine acute hospital and mental healthcare data (Scottish Morbidity Record SMR01 and SMR04) and death certificates to identify episodes of self-harm. The maximum period of follow-up was from 1 January 1981 to 31 December 2012; veterans were followed up from the point of leaving military service if later than 1 January 1981. The data extract was pseudo-anonymised and approval for the study was granted by the Privacy Advisory Committee of the Information Services Division of NHS Scotland.

### Socio-economic status

Socio-economic status (SES) was derived from the most recent registered postcode of residence, using the Scottish Index of Multiple Deprivation (SIMD), in quintiles ranging from 1 (most deprived) to 5 (least deprived). SIMD is calculated on a regional basis, in data zones having a mean population of 800, and is based on income, employment, health, education (including skills and training), housing, crime and access to services [13] to provide a comprehensive measure.

### Definitions

‘Non-fatal self-harm’ was defined as the first occurrence of ICD10 code X60-X84, Y87.0, Y10-Y34 or Y87.2, or ICD9 codes E950-E959 or E980-E959, at any position in the record other than the cause of death, excluding any individual whose death was classified as suicide [4] or whose date of death was the same as the recorded date of self-harm, whilst suicide was classified as the occurrence of any of the specified ICD codes in the cause of death field. ‘Early service leavers’ (ESL) were defined as veterans who had left with 2.5 years’ service or less. Although shorter than the current 4-year minimum period of service, this ensured that veterans who completed the earlier minimum of 3 years’ service were not incorrectly classified as ESL [14]. Veterans having 0.4 years’ service (20 weeks) or less were categorised as not having completed initial training.

### Statistical methods

Cox proportional hazard models were used to examine the association between veteran status and cumulative risk of self-harm, using age as the time-dependent variable, age at first admission for self-harm as the failure time, and age at death (if no self-harm) as the censor time. The use of a time-dependent analysis (Cox proportional hazards) protects against immortal time bias [15, 16]. Cox proportionality assumptions were tested using methodology based on Schoenfeld residuals [17]. The log-likelihood test was used to test for interactions between veteran status, mental health co-morbidity and length of service. The a priori rejection level was set at 0.05. The models were run univariately and then repeated adjusting for SES. The analyses were repeated stratifying by grouped year of birth to examine potential birth cohort effects, and by length of service. The association between suicide and a previous episode of non-fatal self-harm was examined, as were co-morbidities with non-fatal self-harm. All analyses were performed using Stata® v12.1.

## Results

### Main findings

After data cleansing to remove incomplete or invalid records, 56,205 (99.3%) veterans and 172,741 (99.9%) non-veterans were included in the analysis. There were 50,970 (90.7%) male veterans and 5,235 (9.3%) female, reflecting the gender balance of the UK armed forces. The mean period of follow-up was 29.3 years, and there was a total of 6.7 million person years of follow-up among veterans and non-veterans combined. The non-veteran group may have included an estimated 327 people who were Reservist veterans (0.2%).

**Fig. 1:**
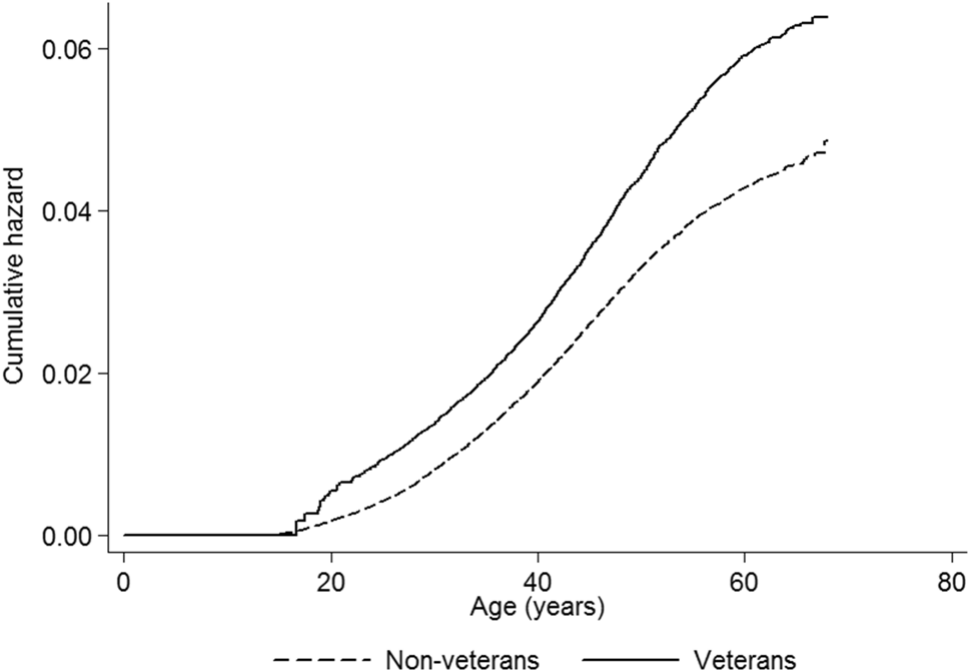
Nelson Aalen plot of risk of non-fatal self-harm in veterans and non-veterans

Overall, 1620 (2.90%) veterans had a record of a first episode of admission to an acute or psychiatric hospital following non-fatal self-harm, compared to 4212 (2.45%) non-veterans. Veterans were at statistically significantly increased risk, unadjusted HR 1.27, 95% CI 1.21–1.35, *p* < 0.001 (Fig. 1). The difference was slightly attenuated after adjusting for SES but remained highly significant, HR 1.20, 95% CI 1.14–1.27, *p* < 0.001. Testing for non-proportionality of the hazards was non-significant, *p* = 0.124. The Nelson-Aalen plot (Fig. 1) demonstrated a sharp increase in risk in the youngest veterans, and a further steep increase in middle age.

### Sex, age, and birth cohort

**Fig. 2:**
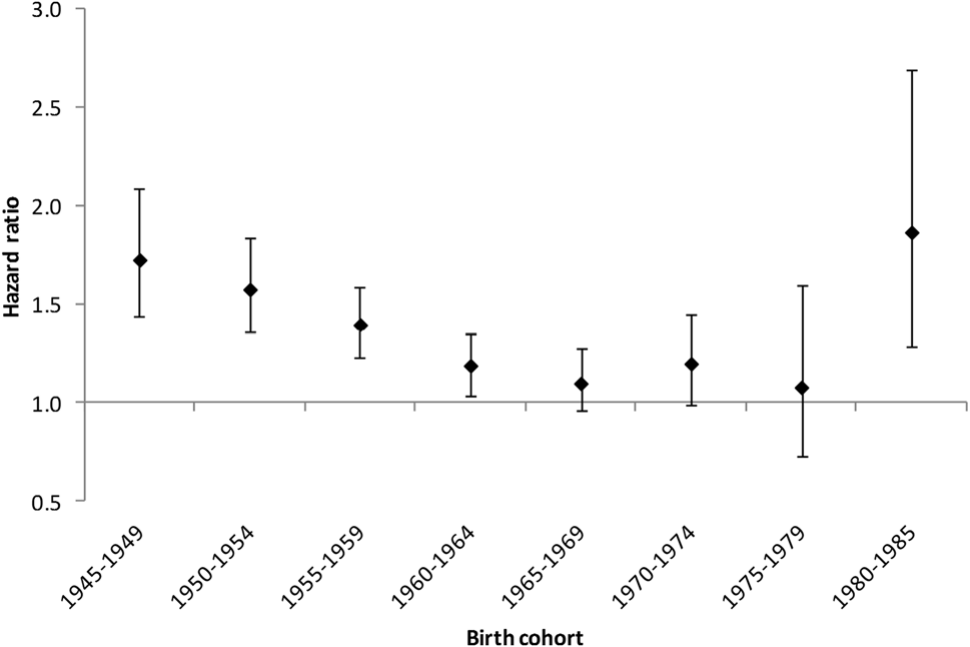
Unadjusted hazard ratios for suicide in veterans referent to non-veterans, by birth cohort

The increase in risk was only significant for men (Table 1); for women, the statistical power was reduced on account of the smaller number of cases, and may have been insufficient to demonstrate a statistically significant difference. The mean age at first episode of self-harm was 41.9 years (SD 9.5) for male veterans, compared with 40.1 years (SD 9.5) for male non-veterans. In women the mean age was 38.9 years (SD 10.1) for veterans and 37.5 years (SD 10.7) for non-veterans. Subgroup analysis by birth cohort showed a U-shaped curve, with the highest risks in both in the oldest (1945–1949) and the youngest (1980–1985) birth cohorts, consistent with the pattern of risk demonstrated by the Cox proportional hazard model. Veterans born between 1965 and 1979 showed no significant difference in risk from non-veterans (Fig. 2).

**Table 1:** Cox proportional hazard model of the association between veteran status and risk of non-fatal self-harm, overall and in two birth cohorts

Veteran cases *n* = *1620*		All Veterans *n*-56,205			Born 1945–1959 *n* = 29,709			Born 1960–1985 *n* = 26,496		
		HR	95% CI	*p*	HR	95% CI	*p*	HR	95% CI	*p*
Overall	1620	1.34	1.26–1.42	< 0.001	1.55	1.42–1.69	< 0.001	1.19	1.10–1.30	< 0.001
Overall (multivariate)	1620	1.26	1.18–1.33	< 0.001	1.43	1.31–1.56	< 0.001	1.17	1.08–1.27	< 0.001
Men	1450	1.38	1.30–1.47	< 0.001	1.61	1.47–1.76	< 0.001	1.23	1.12–1.34	< 0.001
Women	170	1.12	0.94–1.34	0.199	1.23	0.95–1.61	0.120	1.04	0.82–1.32	0.741
Length of Service										
Untrained ESL	259	1.69	1.50–1.91	< 0.001	2.08	1.76–2.45	< 0.001	1.52	1.28–1.80	< 0.001
Trained ESL	480	1.59	1.46–1.74	< 0.001	1.92	1.69–2.18	< 0.001	1.45	1.28–1.65	< 0.001
4–6 years	345	1.33	1.20–1.47	< 0.001	1.58	1.36–1.83	< 0.001	1.19	1.03–1.37	0.018
7–9 years	211	1.19	1.04–1.36	0.008	1.57	1.31–1.88	< 0.001	0.89	0.73–1.08	0.249
10–12 years	128	0.97	0.82–1.14	0.690	1.17	0.93–1.47	0.190	0.77	0.60–0.99	0.044
13–16 years	94	0.97	0.79–1.18	0.740	1.21	0.92–1.58	0.179	0.71	0.53–0.96	0.026
17–22 years	67	0.89	0.70–1.14	0.362	0.87	0.64–1.18	0.358	0.96	0.65–1.41	0.818
≥ 23 years	31	0.40	0.28–0.57	< 0.001	0.41	0.28–0.62	< 0.001	0.38	0.18–0.80	0.011

*HR* hazard ratio, *CI* confidence intervals, *ESL* early service leavers, univariate HR except where stated; multivariate adjusted for SES

### Length of service

Analysis by length of service showed that the increase in risk was almost entirely confined to ESL, with the highest risk in those who did not complete training, unadjusted HR 1.69, 95% CI 1.50–1.91, *p* < 0.001. Older veterans with between 4 and 9 years’ service also showed an increase in risk but when stratified by birth cohort, the risk was only increased beyond 6 years of service in those born before 1960. The risk was reduced, in comparison with non-veterans, in older veterans with over 16 years’ service, although veterans born from 1960 showed a reduction in risk for all lengths of service beyond 6 years. (Table 1).

**Fig. 3:**
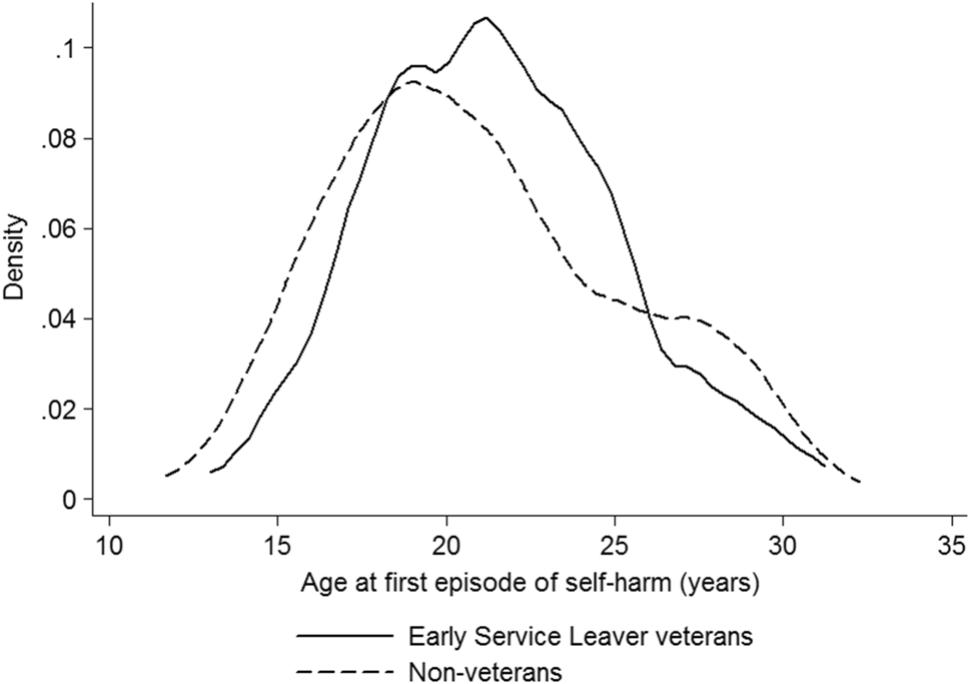
Kernel density plot of age at first episode of non-fatal self-harm, comparing early service leavers and non-veterans, 1980–1985 birth cohort

The kernel density plot for the youngest (1980–1985) birth cohort, comparing age at first episode of self-harm in Early Service Leavers against matched non-veterans, shows that these veterans have fewer pre-service (age 16 and under) episodes but a peak in the early 20s (Fig. 3).

### Association with suicide

We examined data on 266 veterans and 901 non-veterans who died as a result of suicide. Of these, 50 (18.80%) veterans had a record of a previous episode of self-harm, as did 176 (19.53%) non-veterans. The difference was not statistically significant, *p* = 0.860. The median elapsed time between the first recorded episode and the fatal episode was 2.0 years for veterans (IQR 0.4–4.3), whilst for non-veterans it was 2.4 years (IQR 0.5–5.8), although the range extended to 13 years for veterans and 23 years for non-veterans. The mean age at suicide for veterans with a previous history of self-harm was 45.5 years (SD 7.9), compared with 43.6 years (SD 8.1) for veterans with no such history, whilst for non-veterans it was 41.6 years (SD 8.6) compared with 41.1 years (SD 9.8). Only 9% of veterans who died as a result of suicide aged under 30 had a previous recorded episode of self-harm, whilst for veterans aged 50 and over at suicide, the figure was 26%. For non-veterans, the change was less marked and the corresponding figures were 16% and 19%.

### Co-morbidities

Fifty per cent of veterans and 46% of non-veterans who were admitted following self-harm also had a recorded secondary care diagnosis of mental health disorder (mood disorder, anxiety including post-traumatic stress disorder (PTSD), or psychosis), either concurrently or on a separate occasion, compared with 5% of the wider veteran population and 4.5% of the non-veterans (Table 2). Of these, the commonest was mood disorder (37% of veteran cases and 35% of non-veterans). A diagnosis of severe stress or PTSD was recorded in 17% of veterans and 12% of non-veterans who self-harmed. Testing for interaction between severe stress/PTSD and ESL status was highly significant, *p* < 0.001. Physical co-morbidities were common; 13% of both veterans and non-veterans who self-harmed had a history of COPD, compared with 4% of the wider veteran and non-veteran population, whilst 7% had a history of peptic ulcer, approximately twice the proportion in the overall population. Diabetes was also more common in those who self-harmed, as were alcoholic liver disease and hepatitis C, although in the case of the latter two conditions, at a lower level in veterans than in non-veterans. Small numbers precluded analysis of multi-morbidity.

**Table 2:** Comorbdities with non-fatal self-harm in veterans and non-veterans

	Veterans		Non-veterans		Veterans referent to non-veterans		
	Self-harm cases *n* = 1,620 No. (%)	Pop. *n* = 56,205 (%)	Self-harm cases *n* = 4,212 No. (%)	Pop. *n* = 172,71 (%)	OR	95% CI	*p*
Peptic ulcer	128 (7.9)	(3.4)	310 (7.4)	(3.0)	1.07	0.88–1.31	0.482
COPD	209 (12.9)	(4.3)	558 (13.2)	(4.5)	0.97	0.84–1.13	0.726
Diabetes	112 (6.9)	(3.4)	234 (5.6)	(3.3)	1.24	1.00–1.55	0.049
Any cancer	134 (8.3)	(6.4)	290 (6.9)	(6.7)	1.20	0.99–1.46	0.068
AMI	86 (5.3)	(3.7)	183 (4.3)	(3.0)	1.22	0.95–1.57	0.116
Alcoholic liver disease	91 (5.6)	(1.2)	281 (6.7)	(1.3)	0.84	0.67–1.06	0.140
Hepatitis C	25 (1.5)	(0.2)	134 (3.2)	(0.4)	0.49	0.32–0.74	< 0.001
Lung cancer	27 (1.7)	(0.8)	33 (0.8)	(0.6)	2.13	1.28–3.53	0.002
Any mental health disorder	811 (50.1)	(5.0)	1955 (46.4)	(4.5)	1.08	1.02–1.14	0.013
Any mental health disorder *excl. PTSD*	536 (33.1)	(3.9)	1451 (34.4)	(3.8)	0.96	0.89–1.04	0.325
Mood disorder	604 (37.3)	(2.8)	1491 (35.4)	(2.6)	1.05	0.98–1.14	0.179
Anxiety	404 (24.9)	(2.5)	893 (21.2)	(2.0)	1.18	1.06–1.30	0.002
Stress/PTSD	275 (16.9)	(1.1)	504 (11.9)	(0.7)	1.42	1.24–1.62	< 0.001
Anxiety excl. stress/PTSD	129 (9.6)	(1.4)	389 (10.5)	(1.3)	0.91	0.76–1.10	0.351
Psychosis	123 (7.6)	(1.0)	371 (8.8)	(1.1)	0.86	0.71–1.05	0.135

*p* values for odds ratios (OR) comparing crude overall incidence, veterans:non-veterans

## Discussion

We have demonstrated that non-fatal self harm is more common in veterans than in non-veterans matched for age, sex and area of residence, but we have also shown that it is most common in both the oldest and the youngest veterans. The excess risk is restricted to those with the shortest service and is highest in those who left before completing initial training, whilst longer service is protective, suggesting that there is both a ‘healthy worker effect’ [18] and a selection bias effect owing to early discharge of those least suited to military service, who have been shown previously to be at increased risk of mental health disorder [19]. There are two distinct patterns of risk, in the early 20s for the youngest veterans who left service early, and in middle age. We had no clinical details of the cases and were, therefore, unable to distinguish between non-suicidal self-harm and failed suicide; however, it seems likely that the younger ESL cases largely represent the former whereas those presenting in middle age may be more likely to represent failed suicide attempts, based on patterns of deliberate self-harm reported in other studies [20], on the pattern of suicide in veterans that we have previously reported [7], and on the reported occurrence of prior self-harm in relation to completed suicide that we have found in this study.

Although deliberate self-harm and parasuicide (attempted suicide) are frequently considered to lie on the same spectrum, there is good evidence that the two have different underlying psychopathology and should be considered separately, as now recognised by the separate DSM-5 classifications of ‘non-suicidal self-injury’ and ‘suicidal behavior’ [21, 22]. Whilst suicide attempts often involve poisoning with drugs or chemicals, hanging, or jumping from a height, with the clear intention of ending life, deliberate self-harm more often involves disfiguring or painful behaviours of relatively low lethality such as cutting, biting, or mutilation. The behaviour pattern often commences in adolescence, and episodes may occur repeatedly [23]. The lifetime prevalence of self-harm has been estimated to lie between 2.2 and 6% [24], and approximately 4% of a sample of US Air Force recruits were found to have self-harmed [20]. Although self-harming may occur at any age, it is more common in adolescents and young adults. Our finding that suicide in older veterans was more often associated with a previous episode of self-harm than in younger veterans would tend to support the two groups being considered separately in planning preventive strategies.

The literature on gender difference is inconclusive but generally the risk is considered to be higher in women. However a large study in Oxford, UK has provided clarification of any apparent discrepancies by demonstrating a changing ratio with age, with a high female:male ratio for self-harm in adolescents which reverses to give an excess in men beyond the age of 50 [25]. Adverse childhood events, and especially childhood sexual trauma, have consistently been found to be associated with self-harming behaviour [24], and there is a well-documented association with anxiety and depression [20] which is consistent with our finding of a high level of mental health comorbidity.

In a systematic review of 90 prospective studies of people who had self-harmed, Owens et al. found a median 16% repeating non-fatal self-harm within a year, rising to 20–25% after 4 or more years. The risk of subsequent suicide was much lower, 2% at 1 year rising to 7% after 9 years. The authors suggest that recent self-harm is associated with a 1% risk of suicide, although there is a very low positive predictive value for any method of risk assessment for suicide even in groups of patients considered to be at high risk [26, 27]. In a small study of serving Irish military personnel, only 10% of those who died as a result of suicide had a documented history of previous self-harm [28], which is broadly consistent with our finding of 9% in people under 30. Our findings are also supported by the Oxford study which has has demonstrated that the risk of subsequent suicide after self-harm changes with age, being rare in teenagers (less than 1 in 200), but increasing to more than 1 in 10 in people over 60 [29].

In a study of 1986 male and female military recruits, of whom 4% reported a history of self-harm, self-harming behaviour was strongly associated with negative temperament including aggression, mistrust and manipulativeness, as well as disinhibition and a range of personality disorders including borderline and dependent. Many stated that they self-harmed as a means of relieving tension [20]. The association between aggression and self-harm may be an important factor in understanding the excess risk which we have documented. In a study by Rona et al. anger/aggression was positively associated with young age, low rank, being in the Army, combat service and being a veteran [30]. Other factors included childhood adversity and anti-social behaviour, which are prevalent in ESL [31] and are also associated with risk of self-harm [32]. Anger and aggression were strongly associated with PTSD, psychological distress and multiple physical symptoms [30]. The link between premature departure from service and poor long-term mental health outcomes mental has previously been documented [14, 31]. It seems probable that there is an inevitable association between suitability for military service, especially combat service, and aggressive personality traits, which provides some explanation for our findings.

### Strengths and limitations

The strengths of the present study are similar to those described elsewhere [7, 12]. The study was based on a large cohort covering the whole of Scotland with up to 30 years’ follow-up. The records of morbidity and causes of death were taken from acute and psychiatric hospital records and national vital records and are, therefore, likely to be reliable. The use of record linkage to analyse individual level data directly derived from these records allowed a robust cohort study design to be employed. The results were able to be matched or adjusted for potential confounders including sex and SES. It was possible to do subgroup analysis by sex, birth cohort and length of service, enabling a picture of the pattern of self-harm in veterans to be built up.

Limitations of the study include possible loss to follow-up of subjects due to migration away from Scotland, which could not be quantified, and the lack of any follow-up data prior to 1981. As the dataset was derived from demographic, vital record and hospital admissions data, there was no information on individual deployments, combat exposure or individual factors such as childhood events or alcohol use. These may represent potential explanatory variables for which we were unable to analyse the impact, or residual confounding for which we were unable to adjust. No information was available about events which did not come to medical attention or were managed solely in primary care; therefore, only the more severe end of the spectrum of self-harm is represented. We have made the assumption that there were no differences between veterans and non-veterans in respect of the setting in which self-harm was managed, other than the frequency of occurrence. Only the last recorded postcode was available which, for veterans, was necessarily post-service. Therefore, the impact of childhood deprivation could not be assessed. Veterans with Reserve service only could not be identified and were included amongst the non-veterans; this could have had the effect of reducing any observed differences between veterans and non-veterans if they also had an increased risk of self-harm, although as the number of Reservist veterans inappropriately classified as non-veterans is likely to have been small, significant impact on our findings is unlikely.

### Summary

Our findings show that the highest risks of non-fatal self-harm are in veterans with the shortest service, including those who did not complete training, and in the oldest and youngest birth cohorts, whilst veterans who had served for longest are at reduced risk. This pattern is not consistent with a causal role for military service but instead suggests that the risk in early service leavers reflects other vulnerabilities such as adverse childhood events. We have also highlighted an important association between mental health disorder, in particular PTSD, and self-harm in ESL veterans. Although overall, fewer than one-fifth of those who died from suicide had a previous record of self-harm, we have illustrated that this risk is higher in older veterans, suggesting that failed suicide attempts in middle age contribute to the increased risk of self-harm which we observed and underlining the importance of treating every self-harm episode in this older age group as a ‘red flag’. The higher risk of self-harm in the most recent birth cohort warrants further investigation. We recommend that specific inquiry into self-harm should be made in all veterans who present with mental health conditions, and especially those with PTSD who left service prematurely.
